# Optical Coherence Tomography in the UK Biobank Study – Rapid Automated Analysis of Retinal Thickness for Large Population-Based Studies

**DOI:** 10.1371/journal.pone.0164095

**Published:** 2016-10-07

**Authors:** Pearse A. Keane, Carlota M. Grossi, Paul J. Foster, Qi Yang, Charles A. Reisman, Kinpui Chan, Tunde Peto, Dhanes Thomas, Praveen J. Patel, UK Biobank Eye Vision Consortium

**Affiliations:** 1 NIHR Biomedical Research Centre, Moorfields Eye Hospital NHS Foundation Trust and UCL Institute of Ophthalmology, London, United Kingdom; 2 Division of Genetics & Epidemiology, UCL Institute of Ophthalmology, London, United Kingdom; 3 Topcon Advanced Biomedical Imaging Laboratory, Oakland, New Jersey, United States of America; Kobenhavns Universitetshospital, DENMARK

## Abstract

**Purpose:**

To describe an approach to the use of optical coherence tomography (OCT) imaging in large, population-based studies, including methods for OCT image acquisition, storage, and the remote, rapid, automated analysis of retinal thickness.

**Methods:**

In UK Biobank, OCT images were acquired between 2009 and 2010 using a commercially available “spectral domain” OCT device (3D OCT-1000, Topcon). Images were obtained using a raster scan protocol, 6 mm x 6 mm in area, and consisting of 128 B-scans. OCT image sets were stored on UK Biobank servers in a central repository, adjacent to high performance computers. Rapid, automated analysis of retinal thickness was performed using custom image segmentation software developed by the Topcon Advanced Biomedical Imaging Laboratory (TABIL). This software employs dual-scale gradient information to allow for automated segmentation of nine intraretinal boundaries in a rapid fashion.

**Results:**

67,321 participants (134,642 eyes) in UK Biobank underwent OCT imaging of both eyes as part of the ocular module. 134,611 images were successfully processed with 31 images failing segmentation analysis due to corrupted OCT files or withdrawal of subject consent for UKBB study participation. Average time taken to call up an image from the database and complete segmentation analysis was approximately 120 seconds per data set per login, and analysis of the entire dataset was completed in approximately 28 days.

**Conclusions:**

We report an approach to the rapid, automated measurement of retinal thickness from nearly 140,000 OCT image sets from the UK Biobank. In the near future, these measurements will be publically available for utilization by researchers around the world, and thus for correlation with the wealth of other data collected in UK Biobank. The automated analysis approaches we describe may be of utility for future large population-based epidemiological studies, clinical trials, and screening programs that employ OCT imaging.

## Introduction

UK Biobank is a community-based prospective cohort study, currently underway in the United Kingdom (UK), which is unprecedented in terms of both its data collection “breadth” and “depth”.[1–3] In this study, 500,000 participants, aged 40–69 years at enrollment, have been recruited, and will be followed over a period of at least 25 years. For each subject, exhaustive baseline data collection has already been performed based on questionnaires, physical measurements, and biological samples. Questionnaires will assess a range of diverse factors, including general health and disability, socio-demographic profile, smoking/alcohol usage, and dietary habits. Physical measurements included electrocardiography and exercise tolerance, spirometry, and bone density measurement, amongst others. Biological samples collected included blood, urine, and saliva. Using DNA extracted from the blood samples, high throughput genotyping is underway on all 500,000 participants. As such, UK Biobank has the potential to profoundly transform our understanding of the risk factors for disease.[3]

Although not included among the physical measurements from the initial cohort of subjects, a detailed examination of ocular health was later incorporated into UK Biobank.[1, 3] This ocular evaluation included measurements of 1) best-corrected visual acuity, 2) refractive error, and 3) intraocular pressure. Imaging of the eye was also performed, with color photography and optical coherence tomography (OCT). OCT was first described in 1991,[4] and has revolutionized the diagnosis and management of ocular disease.[5] By providing high-resolution cross-sectional (tomographic) images of the neurosensory retina in a completely non-invasive manner, OCT imaging has become indispensable for the assessment of patients with retinal disease, the commonest causes of blindness in the developed world.[6–9] Furthermore, by allowing direct visualization of central nervous system (CNS) tissue and its associated vasculature, retinal imaging with OCT and color photography may provide unique insights into the aging process and into systemic diseases such as those affecting the cardiovascular and neurological systems.[10–12]

A unique advantage of OCT imaging is its extremely high axial resolution–typically 3–8 μm when imaging the retina.[13] Image acquisition is also extremely fast, allowing comprehensive retinal scanning in seconds (typically 100+ macular scans). As a result, OCT imaging has sometimes been described as “*in vivo* clinical biopsy”. Due to its excellent resolution, OCT allows for accurate measurements of thickness of the neurosensory retina.[14–16] OCT is also well suited to visualization of the multi-layered architecture of the retina, and measurement of individual retinal sublayers is possible.[15] In clinical research, OCT image “segmentation” (delineation of boundaries to allow measurements) is often performed manually by trained image graders.[17, 18] While highly accurate, such an approach is time-consuming and therefore not feasible for large studies such as UK Biobank. Automated segmentation algorithms have been developed, although many are inaccurate, slow, and do not allow for batch processing of image sets from large studies.[19] As OCT imaging is increasingly incorporated into large, population-based epidemiological studies, approaches to allow for rapid, automated, quantitative analysis of OCT image sets will become increasingly necessary.

In this report, we describe an approach to the use of OCT imaging in large, population-based studies, including methods for OCT image acquisition, storage, remote analysis, and–most importantly–rapid, automated analysis of retinal thickness.

## Materials and Methods

### Ocular Examination in UK Biobank

Ocular data collection in UK Biobank commenced in September 2009 and involved six study centers around the UK (Sheffield, Liverpool, Birmingham, Croydon, Hounslow, and Swansea). Acquisition of OCT images and retinal photography began in December 2009. No additional eligibility criteria were required for those UK Biobank participants undergoing ocular data collection. The methods and protocol for the ocular examination component of UK Biobank were designed by ophthalmologists from Moorfields Eye Hospital, London, UK. Best corrected visual acuity was measured using logMAR (logarithm of the minimum angle of resolution), refractive error was measured using an autorefractor (Tomey, Japan), intraocular pressure and corneal biomechanics were assessed using an Ocular Response Analyzer (Reichert Technologies, USA). These ocular examinations, plus OCT imaging and retinal photography (see below), were typically performed in around 11 minutes. The North West Multi-centre Research Ethics Committee approved the study (REC Reference Number: 06/MRE08/65), in accordance with the principles of the Declaration of Helsinki. Written, informed consent was obtained for all participants in UK Biobank.

### Optical Coherence Tomography Image Acquisition and Training

**Fig 1:**
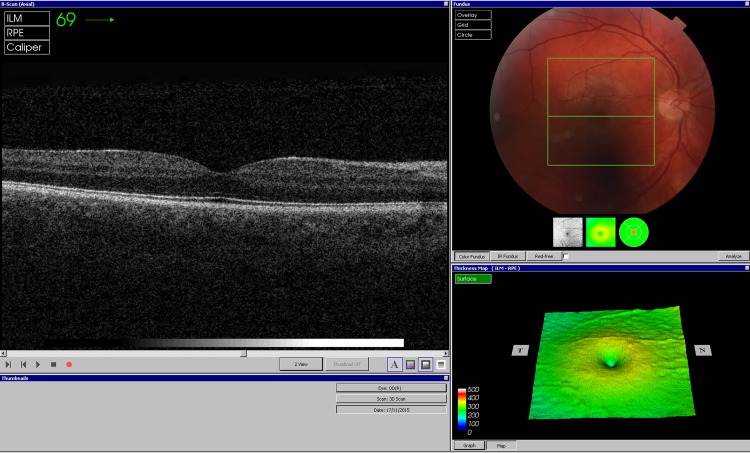
Optical coherence tomography (OCT) image sets. OCT image sets were obtained using a raster scan protocol on a spectral domain OCT system (3D OCT-1000 Mark II, Topcon, Japan).

OCT images were acquired using a commercially available “spectral domain” OCT device (3D OCT-1000 Mark II, Topcon, Japan). This system has an axial resolution of 6μm and an image acquisition speed of 18,000 A-scans per second (each A-scan is the measurement of the reflectance profile along the optical axis within the retina). OCT images were obtained using a raster scan protocol, 6 mm x 6 mm in area, centered on the fovea. This raster scan consisted of 128 B-scans, each composed of 512 A-scans (a B-scan is a two-dimensional, cross-sectional image of retinal tissue) (Fig 1). Using this protocol, a whole macular 3D volume of 512 A-scans by 128 B-scans is obtained in 3.6 seconds (512*128/18000). A very small galvanometer overhead time to complete the image acquisition is also required, leading to a total image acquisition time of 3.7 seconds.

The 3D OCT-1000 system also incorporates a digital camera to allow acquisition of color photographs of the ocular fundus (posterior pole images centered on the macula but including the optic disc).

A training program was developed as a collaboration between the UK Biobank training team (for consistency with other UK Biobank protocols) and by the Moorfields Eye Hospital Reading Centre (MEHRC) (for eye and imaging related knowledge). The approach to training followed the approach taken for other UK Biobank data modules with a focus on practical steps needed to acquire an OCT scan. All personnel selected were either already involved in, or subsequently trained in, other aspects of UK Biobank workflow. No pre-requisite qualifications were required for the eye component training. Training on the components of the ocular module (visual acuity testing, auto-refraction, intraocular pressure measurement) focused on the practical elements needed to be applied in a step-wise manner to acquire the data using standard operating procedures or instructions and all technicians had to pass a structured exam to enable them independently carry out these tests

In addition, UK Biobank technicians working on OCT image acquisition underwent a structured training program and competency exam during which they had to demonstrate that they read and understood the standard operating procedure for OCT image acquisition and demonstrated the ability to acquire well centered images with good signal strength. Once certified, all images from the first day of independent images were quality controlled by the MEHRC ophthalmologists (D.T. and T.P.), and an UK Biobank site duty manager, to resolve any questions or difficulties during the initial phase of independent image acquisition. An additional approximately 10% of the OCT images were also assessed for quality by certified OCT graders at MEHRC. Re-training was provided on any issues that proved less than ideal during the real-time quality assurance review.

Once able to take the images competently, further training focused on pattern recognition to allow the technician to recognize: 1) significant artifactitious variations in signal intensity across the image (generally a sign of irregular media opacity or poor mydriasis, 2) artifactitious severe anomalies in retinal contour (generally a sign of severe refractive error, and 3) generalized reductions in OCT signal strength. This enabled the technician to immediately recognize image acquisition problems and act on these while the subject was still attending the Biobank site. Training was performed by a UK Biobank Trainer and an MEHRC-trained ophthalmologist.

On average, at any given time of the study, a minimum of three examiners per site worked as trained and certified UK Biobank Ophthalmic Technicians. The staff were multi-skilled for ocular and non-ocular assessments and were able to move between stations when required to increase efficiency and prevent delay in the flow through the patient pathway. This process was controlled by a "floor manager" who monitored the patient's progression through the assessment pathway via a USB key carried by the patient. This person was able to re-assign staff to different areas using a strategy not dissimilar to that used in supermarkets where staff are utilized for floor tasks and check out points. There was a minimal turnover of personnel during the study, but there was a mechanism in place to ensure that trained operators were always available at every UK Biobank site. There was never a day when patients could not be imaged due to lack of trained operator or when patients were imaged by an untrained operator.

### Data Monitoring and Quality Assessment Feedback

Custom software was created by the Clinical Trials Service Unit at the University of Oxford to allow for live, ongoing data monitoring during the OCT image acquisition period using electronic direct data entry case reports forms. Grading of OCT image quality was performed on electronic case report forms (CRF). On each CRF, the visual acuity and refractive error were automatically imported and the grader assessed each image set for overall image quality, image focus and centration relative to the fovea, and central macular thickness and accuracy of measurements. In the event of image error, its possible source was attributed to one of the following categories: 1) participant, 2) operator, 3) equipment, or 4) indeterminate. Quality assessment feedback was then provided to each center on an ongoing basis.

### Image Storage and Remote Access

**Fig 2:**
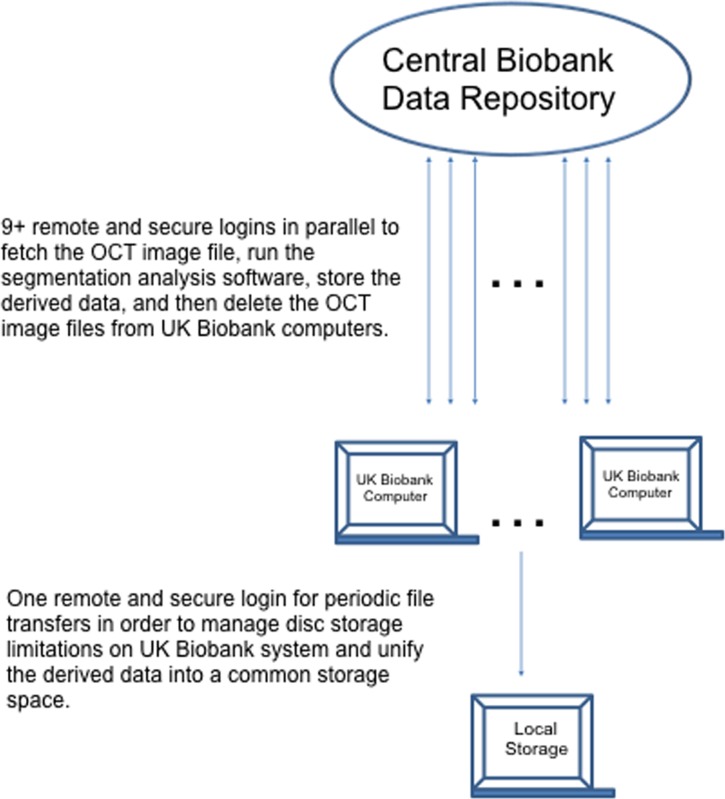
UK Biobank Data Processing Scheme. The source data (optical coherence tomography (OCT) image sets) were stored on a central repository and accessed via remote, secure login.

OCT image sets were stored on UK Biobank servers in a central repository at Advanced Research Computing, University of Oxford (previously known as Oxford Supercomputing Centre (OSC)), adjacent to high performance computers. This consists of: 1) a couple of 1000-core Linux servers, 2) an Nvidia graphics processing unit (GPU) cluster, and 3) a Windows 2012 server which creates and manages a collection of Windows XP/Windows Vista/Windows 7 virtual machines. At the time of our initial analyses, UK Biobank data access rules and procedures for bulk data prohibited copying, storage or removal of OCT files (source data) outside of the Oxford computing system. Instead, researchers were given access to computers at the central repository via remote, secure login and can then install any analysis software needed. A copy of the stored OCT image file is fetched before execution of the segmentation analysis software (see below). The derived data are then extracted, after which the OCT image file is deleted. Multiple logins can be implemented in parallel, increasing the processing throughput (Fig 2).

### Automated Analysis of Retinal Thickness

**Fig 3:**
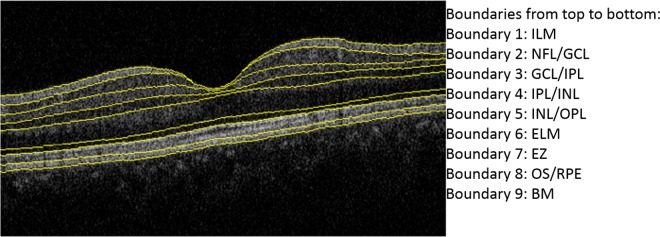
Automated Segmentation of Optical coherence tomography (OCT) image sets. Topcon Advanced Boundary Segmentation (TABS^TM^) software was used to perform automated segmentation of nine intraretinal boundaries. in a rapid fashion. Boundary 7 has previously been described as the inner aspect of the photoreceptor inner segment-outer segment junction (and is still described as this by Topcon Inc.); however, in a recent proposed nomenclature for classification of retinal layers on OCT, this boundary is referred to as the photoreceptor ellipsoid zone.[22]

Rapid, automated analysis of retinal thickness was performed using custom image segmentation software developed and validated by the Topcon Advanced Biomedical Imaging Laboratory (TABIL) (New Jersey, United States). This software, called Topcon Advanced Boundary Segmentation (TABS^TM^), employs dual-scale gradient information to allow for automated segmentation of the inner and outer retinal boundaries, and retinal sublayers, in a rapid fashion (generally less than 60 seconds per raster scan in the UK Biobank analysis using multi-threaded implementation) (Fig 3). The location of the fovea within the scan volume was also automatically determined, allowing for centered sector grid placement. The accuracy and reproducibility of this software has previously been reported,[20] as has its use in a cohort of 256 healthy subjects.[21]

A number of quality control indices were also employed in an effort to highlight and exclude cases with segmentation error. These included an image quality score, an internal limiting membrane (ILM) indicator, a validity count, and motion indicators. The ILM indicator is a measure of measure of minimum localized edge strength along the ILM boundary across the entire scan. It is useful for identifying blinks and segmentation errors. The validity count index is used to identify scans with a significant degree of clipping in the OCT B-scan’s Z-axis direction. Finally, the motion indicators assess the correlation between retinal nerve fiber layer thickness and total retinal thickness, across consecutive B-scans. This last indicator helps to identify blinks, eye motion artifacts and segmentation failures. A more detailed description of these indices is described elsewhere.[23]

## Results

### OCT Image Acquisition

67,321 participants (134,642 eyes) in UK Biobank underwent OCT imaging of both eyes as part of the ocular module. The mean age (± standard deviation (SD)) of patients was 57 (±8) years, with 36,623 females and 30,698 males. OCT image acquisition was completed in six centers across the UK beginning in December 2009.

### OCT Data Size

A single Topcon 3D-OCT Mark-II macular 3D volume has a file size of 97.8MB. The OCT scan data therefore had a total data size in excess of 10TB for the initial round of UK Biobank work. In addition, the computed segmentation and measurement data increased this total by approximately one percent.

### OCT Image Analysis

A total of 134,642 macular OCT images were available for processing from the 134,642 eyes that underwent OCT scanning. Of these images, 134,611 images were successfully processed with 31 images failing segmentation analysis due to corrupted OCT files or withdrawal of subject consent for UKBB study participation. Therefore, successful automated analysis of retinal thickness was obtained for 99.98% of all OCT images acquired.

The time taken to fetch each data set from the database was approximately 70 seconds. The time taken to segmentation analysis was approximately 58 seconds. Therefore, the entire process for each image set was typically completed in 128 seconds. By utilizing multiple logins in parallel, the effective throughput was up to 11 times greater (12 logins minus one which was used largely for data transfers) than these per-login times. As a result, the whole analysis was completed in 28 days. It should also be noted that the 28 days here were not completely efficiently executed, as pauses were intentionally inserted in the batch processes to ensure that the limited shared disk space provided by UK Biobank did not reach capacity. If there had been no pauses whatsoever (i.e., 100% efficiency using 11 login resources), then the entire process would have taken only 18 days. This implies that our execution efficiency was approximately 65%, leaving room for some degree of improvement.

The average signal strength (Q factor) for all images was 65 (±13). Signal strength and other quality indicator. As described above, quality control indicators were applied to highlight and exclude image sets with segmentation error. Use of these indicators led to the exclusion of 15,177 patients. The remaining subset of 51,978 patients had good quality, well-centered images and central, stable fixation during their OCT scan.

## Discussion

In this report, we describe methods used for the acquisition, storage, and remote, automated analysis of OCT image sets from the UK Biobank study. Our approach provides rapid, non-invasive, quantitative measures of retinal thickness (including measures of individual retinal sublayers) for a large population based cohort involving >100,000 eyes. To our knowledge, this is the first study that involves quantitative analysis of OCT images sets on this scale. By comparison, the Beaver Dam Eye Study has recently reported the results of spectral domain OCT imaging; this was also performed with the Topcon 3D-OCT system, but only involved 1544 individuals, and did not include measurements of retinal sublayers.[24] The Beijing Eye Study has also included spectral domain OCT imaging, but with the Heidelberg Spectralis system and involving 3468 individuals. In this study, measurements of subfoveal choroidal thickness were obtained manually using a calipers.[25]

We present these methods in isolation from the specific retinal thickness results for a number of reasons. Firstly, UK Biobank is an open-access resource that encourages researchers from around the world–including those from the academic, nonprofit, public, and commercial sectors–to access the data and biological samples for any health-related research that is in the public interest.[2] As such, the retinal thickness measurements provided by our study will be incorporated back into the resource and made publically available so that others can evaluate their significance as risk factors for disease. Secondly, we believe that our approach has implications for ongoing and future studies incorporating OCT imaging, whether they be large population-based epidemiological studies, phase IV or phase V clinical trials, “real-world” outcome studies, or national screening programs for ocular and systemic disease. For example, the use of electronic medical record (EMR) systems offer the ability to capture and pool a large proportion or even all data from patients undergoing a specific treatment.[26] Such systems have the benefit that all data can be collected as a by-product of routine clinical practice and can be designed to mandate capture of defined minimum datasets. Consequently, they offer a unique opportunity to assess how clinical trial results translate into “real-world” outcomes. In the recent UK Neovascular Age-Related Macular Degeneration (AMD) Database study, use of an EMR allowed assessment of visual outcomes following 92,976 treatments with ranibizumab for this condition.[27, 28] In almost all cases, OCT imaging was obtained at each treatment episode. However, without a method for automated analysis this vast quantity of clinically relevant information is not easily accessible. Similarly, our approach may be of use for screening programs for diseases such as diabetic retinopathy, where OCT is increasingly being incorporated.[29] At present, this typically involves manual assessment of images by trained “graders”–an approach that is expensive, time-consuming, subjective, and often only semi-quantitative. Without the use of rapid, automated OCT analysis techniques, such an approach may not be feasible for inclusion in screening programs on a national scale.

In our study, OCT image sets provided cross-sectional images of the neurosensory retina in the macular region, covering a 6 x 6 mm^2^ area of each participant’s eye. By allowing detailed quantitative analysis of individual retinal sublayers, OCT imaging may thus be of considerable value for the assessment of systemic disease in epidemiological studies. For example, reductions in the thickness of the retinal nerve fiber layer (RNFL) have recently been reported in patients with mild cognitive impairment, Alzheimer’s disease, and Parkinson’s disease.[30, 31] Interestingly, in patients with multiple sclerosis, RNFL thinning appears to correlate with atrophy in both white matter and deep gray matter structures as visualized by magnetic resonance imaging (MRI).[32] In addition to ocular and neurological disease, OCT may be useful for the study of cardiovascular, metabolic, and endocrine disease–in patients with diabetes mellitus, for example, preliminary evidence from small studies suggests that neurodegeneration may precede vascular degeneration.[33, 34] We specifically highlight these medical specialties as, in May 2014, UK Biobank began a multimodal imaging extension study in 100,000 participants. This study will encompass MRI scanning of the brain, heart, and abdomen, carotid artery ultrasonography, and whole-body dual-energy x-ray absorptiometry (DXA) of the bones and joints (http://imaging.ukbiobank.ac.uk, accessed October 1^st^, 2014). Correlation of these findings with OCT measures of retinal thickness is likely to be of particular interest.

While the opportunities afforded by current OCT technology are numerous, they likely represent only the tip of the proverbial iceberg. Since its initial description in 1991,[4] and even since its utilization in UK Biobank in 2009, OCT technology has continued to evolve rapidly.[35] Commercially available OCT systems now allow cross-sectional imaging of the choroid, a tissue with the highest vascular flow rate in the human body.[36–38] The choroidal circulation lacks the autoregulation of the retinal circulation and thus choroidal thickness may be affected by factors such as age,[39] refractive error,[40] diurnal variation,[41] inflammatory disease,[42] renal disease,[43] and numerous medications.[44, 45] Such variability is likely to be of value when studied in large, cross-sectional epidemiological studies. The approaches to automated analysis of retinal thickness described herein have already been modified to incorporate automated measures of choroidal thickness in newer OCT systems.

Recent commercial OCT systems also demonstrate greatly increased image acquisition speed, providing new capabilities such as “widefield” imaging of the ocular fundus (e.g., 12 x 9 mm^2^ in area or greater, incorporating the macula and optic nerve regions in a single scan), and so-called “OCT angiography”, allowing non-invasive mapping of the retinal and choroidal vasculature.[46–48] Recently developed high-speed (100KHz or higher scan rate) OCT systems employ wavelength tunable “swept source” lasers as their light source.[35] The first commercially available swept source OCT system is the DRI OCT-1 Atlantis from Topcon. Swept source lasers are also small and robust lasers and may thus allow future OCT devices to become more readily portable, and even handheld.[49] The adoption of “binocular” designs may further remove the need for additional personnel to acquire OCT by enabling patients to align the optical axes of the instrument with the optical axes of their own eyes.[50] The potential cost-saving implications for large epidemiological studies are clear.

Our approach to automated analysis of OCT image sets has a number of potential limitations and caveats. Although the accuracy and reproducibility of the analysis software has previously been reported in patients with glaucoma, and in healthy volunteers of varying ages, it is less likely to produce accurate results in the presence of ocular disease where there is complex morphological disturbance of the retina (e.g., in patients with advanced neovascular AMD).[20] In such cases, manual segmentation of images at an OCT image-reading center, or using a crowd-sourced approach,[51] is likely to be required. Of note, UK Biobank did not specifically exclude patients with macular disease and this will have affected the accuracy of retinal boundary detection in a proportion of imaged eyes. Another limitation to consider is that although automated segmentation was completed in over 99% of eyes, this should not be confused with accuracy of automated retinal and sublayer boundary detection. Segmentation accuracy depends on a variety of factors including image quality and indeed the prevalence of morphological abnormalities in the sample of OCT images analyzed. In the UK Bioabnk OCT images we excluded 22% of the sample based on indicators of segmentation accuracy when reporting and analyzing retinal thickness in the cohort. In addition, our algorithm provides measures of retinal sublayer thickness but does not provide measures of other morphologic features that may be present as a result of retinal fluid exudation, hemorrhage, or scarring. Again, manual image analysis is likely to be required to achieve this aim.[52] Efforts are underway to develop algorithms that allow for automated detection of ocular diseases, and which place less emphasis on direct measurements of retinal thickness.[53] These algorithms may facilitate selection of those image sets most likely to require reading center grading in large studies. A further limitation of our approach is that the software program employed for this study was OCT system specific (i.e., it is only capable of performing automated analysis of OCT images from the Topcon OCT system). However, the principle of utilizing dual scale gradient information is not OCT vendor specific, and studies are underway utilizing updated versions of the software to perform automated analysis of both Spectralis OCT (Heidelberg Engineering) and Cirrus OCT (Carl Zeiss Meditec) datasets.

## Conclusion

In conclusion, we report an approach to the rapid, automated measurement of retinal thickness from OCT images in the UK Biobank study. Analysis of images from ~140,000 eyes was completed in an entirely automated fashion over a 28 day period. Measurements for the neurosensory retinal thickness as whole, and for individual retinal sublayers, were obtained. In the near future, these measurements will be publically available for utilization by researchers around the world, and thus for correlation with the wealth of other data collected in UK Biobank. Finally, the automated analysis approaches we describe may be of utility for future large population-based epidemiological studies, clinical trials, and screening programs that employ OCT imaging.
